# The socioeconomic profile of alcohol-attributable mortality in South Africa: a modelling study

**DOI:** 10.1186/s12916-018-1080-0

**Published:** 2018-06-25

**Authors:** Charlotte Probst, Charles D. H. Parry, Hans-Ulrich Wittchen, Jürgen Rehm

**Affiliations:** 1 Institute for Mental Health Policy Research, Centre for Addiction and Mental Health (CAMH), 33 Russell Street, Toronto, ON M5S 2S1 Canada; 2 Institute for Clinical Psychology and Psychotherapy, Technische Universität Dresden, Chemnitzer Str. 46, 01187 Dresden, Germany; 3 Alcohol, Tobacco and Other Drug Research Unit, South African Medical Research Council, Cape Town, Tygerberg 7505 South Africa; 4 Department of Psychiatry, Stellenbosch University, Cape Town, Tygerberg 7505 South Africa; 5 Research Group Clinical Psychology and Psychotherapy, Psychiatric University Hospital, Ludwig-Maximilians-University, Nußbaumstraße 7, 80336 Munich, Germany; 6 Addiction Policy, Dalla Lana School of Public Health, University of Toronto, 155 College Street, Toronto, ON M5T 3M7 Canada; 7 Institute of Medical Science, University of Toronto, Faculty of Medicine, Medical Sciences Building, 1 King’s College Circle, Toronto, ON M5S 1A8 Canada; 8 Department of Psychiatry, University of Toronto, 250 College Street, Toronto, ON M5T 1R8 Canada

**Keywords:** Alcohol consumption, Mortality, Burden of disease, Inequalities, Socioeconomic status, South Africa

## Abstract

**Background:**

Globally, illness and life expectancy follow a social gradient that puts people of lower socioeconomic status (SES) at higher risk of dying prematurely. Alcohol consumption has been shown to be a factor contributing to socioeconomic differences in mortality. However, little evidence is available from low- and middle-income countries. The objective of this study was to quantify mortality attributable to alcohol consumption in the adult (15+ years) general population of South Africa in 2015 by SES, age, and sex.

**Methods:**

A comparative risk assessment was performed using individual and aggregate data from South Africa and risk relations reported in the literature. Alcohol-attributable fractions (AAFs) and alcohol-attributable mortality rates were estimated for cause-specific mortality by SES, sex, and age. Monte Carlo simulation techniques were used to calculate 95% uncertainty intervals (UI).

**Results:**

Overall, approximately 62,300 (95% UI 27,000–103,000) adults died from alcohol-attributable causes in South Africa in 2015, with 60% of deaths occurring in people in the low and 15% in the high SES groups. Age-standardized, alcohol-attributable mortality rates per 100,000 adults were highest for the low SES group (727 deaths, 95% UI 354–1208 deaths) followed by the middle (377 deaths, 95% UI 165–687 deaths) and high SES groups (163 deaths, 95% UI 71–289 deaths). The socioeconomic differences were highest for mortality from infectious diseases.

People of low SES had a lower prevalence of current alcohol use but heavier drinking patterns among current drinkers. Among men, AAFs were elevated at low and middle SES, particularly for the middle and higher age groups (35+). Among women, AAFs differed less across SES groups and, in the youngest age group (15–34), women of high SES had elevated AAFs.

**Conclusions:**

Alcohol use contributed to vast socioeconomic differences in mortality. Where observed, elevated AAFs for people of low and middle SES arose from higher levels of consumption among current drinkers and not from the prevalence of current alcohol use per se. The findings can direct preventive measures and interventions on those at highest risk. Future research is needed to investigate socioeconomic differences in the risk functions relating alcohol use to cause-specific mortality.

**Electronic supplementary material:**

The online version of this article (10.1186/s12916-018-1080-0) contains supplementary material, which is available to authorized users.

## Background

Globally, illness and life expectancy follow a social gradient that puts populations of poorer countries [1, 2], as well as persons of lower socioeconomic status (SES) within a country, at higher risk of dying prematurely [3, 4]. Alcohol consumption has been found to contribute to the socioeconomic differences in mortality within countries [5, 6]. However, most of this research has focused on high-income countries, and thus may not apply to the situation of low- and middle-income countries such as South Africa.

The latest estimates by the World Bank showed that, in 2010, over half of the South African general population lived below the national poverty line, despite South Africa being an upper middle-income country [7]. However, with a Gini coefficient of above 60, South Africa is among the countries with the highest inequalities in income and wealth worldwide [7]. Not least due to the history of colonialism and apartheid, socioeconomic differences in South Africa are heavily intertwined with race [8].

In 2016, only approximately 20% of women and 50% of men were current drinkers [9]. However, the prevalence of heavy drinking occasions among the current drinkers was found to be high, with 20% among women and 45% among men. Furthermore, per capita consumption in South Africa in 2010 was almost double that of the regional average, indicating high levels of consumption among current drinkers [10].

Socioeconomic inequalities in health and mortality have been found within countries around the world [3]. Nonetheless, studies rarely went beyond quantifying relative or absolute measures of inequality in (all-cause) mortality between the socioeconomic groups [3, 11, 12] or reporting cause-specific deaths in socioeconomic strata without consideration of alcohol exposure beyond the investigation of fully alcohol-attributable causes of death such as alcoholic liver cirrhosis [6, 13]. Current comparative risk assessments, on the other hand, quantify the impact of risk factors in subgroups defined by age and sex but not for distinct socioeconomic strata [14, 15].

The objective of the present study was to investigate the mortality attributable to alcohol use in different socioeconomic groups in South Africa. Specifically, the aim was to estimate alcohol-attributable fractions (AAFs) and mortality rates attributable to alcohol consumption stratified by SES, sex, and age in South Africa in 2015. It was expected that alcohol use contributes to the socioeconomic differences in mortality, with elevated AAFs among persons of lower SES.

## Methods

### Study design

**Fig. 1:**
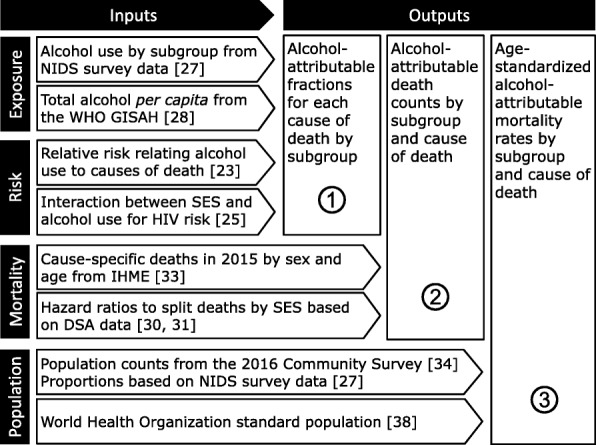
Schematic overview of the data sources and relative risks used in the calculation steps. A subgroup was defined by socioeconomic status, age, and sex. *DSA* Demographic Surveillance Area, *GISHA* Global Information System on Alcohol and Health, *IHME* Institute for Health Metrics and Evaluation, *NIDS* National Income Dynamics Study, *SES* socioeconomic status, *WHO* World Health Organization

A comparative risk assessment was performed for the adult (15+ years) general population of South Africa in the year 2015, using individual data, aggregate data, and risks relations reported in the literature [16, 17]. A schematic overview over the data sources used in the three main steps of calculation is shown in Fig. 1.

All calculation steps were performed for sociodemographic subgroups defined by SES, age, and sex. SES was measured through an asset score [18–20], incorporating information on ownership of household assets such as a refrigerator, TV, or car as well as access to water, sanitation, and electricity. Asset scores were calculated using Multiple Correspondence Analysis [21, 22], resulting in three approximately equally sized groups representing high, middle, and low SES. Details on the calculation of asset scores are presented in the Additional file 1. Age was measured in 5-year age groups for all metrics of mortality and 20-year age groups (15–34, 35–54, and > 55 years) for exposure to alcohol and the respective AAFs.

Exposure to alcohol was categorized into lifetime abstinence (never used alcohol), former drinking (no alcohol consumption in the past 12 months), current drinking, and binge drinking (5+ standard drinks per usual occasion). The level of exposure was quantified in average grams of pure alcohol per day among current drinkers. Risk relationships between exposure to alcohol and cause-specific mortality were taken from Rehm et al. [23] (see also [24]). Based on the results of a large representative study on South Africa [25], an interaction effect was modeled for testing HIV positive in a way that, in addition to the main effects of current alcohol use and SES, an interaction variable for alcohol use * SES was added to the equation. This interaction effect was accounted for in the current comparative risk assessment, leading to an increased relative risk for current drinkers of low SES when calculating the AAFs. For more details on the implementation see Probst et al. [26].

In total, 19 cause of death categories were investigated, including communicable diseases, namely HIV/AIDS, lower respiratory infections, and tuberculosis; non-communicable diseases such as ischemic heart disease, hemorrhagic stroke, or diabetes; and all injuries (for details see Additional file 1: Table S1). Causes of death were selected to include all major causes of death for which alcohol use has been shown to be a causal risk factor [23].

### Data sources

Secondary, individual-level data on alcohol consumption in each sociodemographic subgroup came from the National Income Dynamics Study (NIDS) [27]. The survey was conducted from October 2014 to August 2015, with an overall response rate of 86.5%. After removing 4078 observations with missing data on age, sex, SES, alcohol use, or survey weights, the sample comprised 22,741 adults (15+ years) with complete data. Although the survey was designed to be nationally representative, given the household sampling frame, some populations, such as homeless people, were excluded (for details see Additional file 1).

Alcohol use was assessed as usual frequency (ranging from never to daily) and quantity (number of standard drinks per occasion ranging from 1 or 2 to 13 or more). Asset variables and the generation of the asset score using NIDS data are described in Additional file 1.

The aggregated total adult per capita consumption in liters of pure alcohol in 2015 (9.5 L) was obtained from the World Health Organization (WHO) Global Information System on Alcohol and Health [28]. In order to account for possible underreporting in medical epidemiological studies and for alcohol that was produced and sold but not consumed, 80% of the total adult per capita consumption was used [29].

Cohort data containing information on cause of death, age, sex, and SES were obtained from the Demographic Surveillance Area (DSA) of the Africa Centre for Health and Population Studies for the years 2000 to 2014 [30, 31]. The open cohort covering approximately 11,000 households was based on a full assessment of a predominantly rural area in the Umkhanyakude district of KwaZulu-Natal, comprising also periurban informal settlements and a township. After removing 923 participants for which at least one of the relevant variables were missing, the sample comprised 87,029 adults, 757,404 person-years and 23,002 deaths. Each household was visited at least twice a year. The cause of death was established using verbal autopsies [32] and information on household assets and basic services was obtained in face-to-face interviews. The earliest observation with information on household assets was used as baseline asset score. Details on generating the asset score are described in Additional file 1. In order to correct for the predominantly rural population of the study area of the DSA data, the asset score was ‘projected’ onto nationally representative data. This was done by sub-setting the national survey data to rural and periurban areas of Kwazulu-Natal and using the percentiles that corresponded to the tertiles of the national distribution as cut-offs to split the asset score of the DSA data [26].

Aggregated death counts on the national level in 2015 by cause of death, 5-year age group, and sex were obtained from the Institute for Health Metrics and Evaluation [33]. Population counts were based on estimates from the Community Survey 2016 [34].

### Data analysis

Step 1: AAFs represent the proportion of deaths that would not have occurred in the hypothetical counterfactual scenario of everyone being a lifetime abstainer. AAFs were calculated by combining the level of exposure to alcohol with the cause-specific mortality risk related to the exposure [29].

Measures of exposure to alcohol by sociodemographic subgroup were calculated using NIDS data. The average grams of pure alcohol per day among current drinkers were calculated using the standard quantity-frequency approach [35]. In order to account for the underestimation of the grams of pure alcohol per day based on survey data [36], information on the relative quantity consumed by each sociodemographic subgroup (based on NIDS data) were combined with the aggregated total adult per capita consumption in liters of pure alcohol (taken from the WHO Global Information System on Alcohol and Health). The standard triangulation technique of the Global Burden of Disease study and WHO Global Status Reports was used [29, 37].

Step 2: AAFs were applied to cause-specific death counts in each subgroup. As the aggregate Institute for Health Metrics and Evaluation death counts were only available by age and sex, hazard ratios (HRs) were used to split death counts in each subgroup by SES. The HRs were derived from DSA data using Cox proportional hazards survival analyses for specific causes of death as well as broader cause categories, adjusting for age at baseline and sex. A sensitivity analysis was performed to examine the stability of the results when using a simple tertile-split asset score for calculating HRs (i.e., without projection onto the national distribution). Details on the HRs are shown in Additional file 1: Table S2.

Step 3: In order to calculate alcohol-attributable mortality rates per 100,000 adult population, the death counts in each subgroup were divided by population counts. Age- and sex-specific population counts were split by SES using the proportions observed in the nationally representative NIDS data. Death rates were age-standardized using the WHO reference population [38].

Uncertainty of AAFs and mortality rates was estimated using Monte Carlo simulations, sampling from the distribution of each lowest level parameter 100,000 times [39]. The 95% uncertainty interval (UI) was determined using the 2.5th and 97.5th percentile of the resulting distribution of the metric. Taking the uncertainty as expressed in the standard error of all lowest level metrics that enter the calculation of the final estimate into account, the true point estimate would fall into this range 95% of the time. This, of course, relies on the assumption that the underlying lowest level metrics follow the assumed (usually normal or binomial) distribution.

## Results

### Alcohol consumption and AAFs

The prevalence of current drinking decreased from high to low SES, and persons of lower SES were more likely to be lifetime abstainers (Table 1). However, the grams of pure alcohol consumed among current drinkers decreased from low to high SES and the prevalence of binge drinking was highest in the middle SES group, followed by the lowest SES group, particularly among men.

**Table 1:** Population statistics on alcohol consumption and mortality by sex and socioeconomic status, South Africa, 2015

	Men			Women		
SES	High	Middle	Low	High	Middle	Low
Population (15+ years)	5,838,935	6,030,496	6,305,738	7,081,701	6,828,503	6,782,163
Lifetime abstainers in % (95% CI)	35.4 (32.0–38.9)	36.7 (34.1–39.4)	40.8 (38.2–43.3)	58.6 (54.5–62.7)	70.8 (67.8–73.7)	76.6 (74.2–79.0)
Current drinkers in % (95% CI)	50.5 (46.0–55.0)	47.8 (44.5–51.2)	45.0 (41.9–48.1)	28.5 (24.4–32.7)	17.8 (15.4–20.1)	13.9 (12.0–15.8)
Binge drinkers in % (95% CI)	11.9 (9.5–14.4)	14.7 (12.3–17.1)	13.5 (11.4–15.6)	2.6 (1.7–3.4)	2.9 (2.1–3.8)	2.7 (1.9–3.6)
Mean grams per day among drinkers (SD)	47.7 (55.9)	65.6 (76.8)	67.4 (78.9)	20.3 (25.6)	29.4 (36.9)	41.6 (52.3)
Total deaths^a^ (95% UI)	48,469 (43,512–53,930)	95,873 (82,905–108,684)	122,436 (108,590–135,061)	44,289 (39,589–49,428)	92,189 (79,384–104,727)	126,146 (112,328–138,705)
Deaths attributable to alcohol consumption by broader categories (95% UI)						
Injuries	1850 (1223–2593)	4334 (2733–6247)	4600 (2975–6489)	320 (206–464)	604 (365–896)	627 (383–922)
Infectious diseases	2051 (646–3804)	4768 (1541–8924)	18,311 (5472–32,176)	601 (178–1324)	1171 (331–2732)	6520 (1558–14,572)
Chronic diseases	2985 (1007–5486)	4205 (1470–7982)	5981 (2731–10,110)	1181 (− 126–3333)	1946 (175–5142)	3240 (642–6950)
Total	6886 (2876–11,883)	13,307 (5744–23,153)	28,892 (11,178–48,775)	2102 (258–5121)	3721 (871–8770)	10,387 (2583–22,444)

*SES* socioeconomic status, *CI* confidence interval, *UI* uncertainty interval

^a^All deaths that occurred among adults (aged 15+) in South Africa in 2015, split by SES

**Fig. 2:**
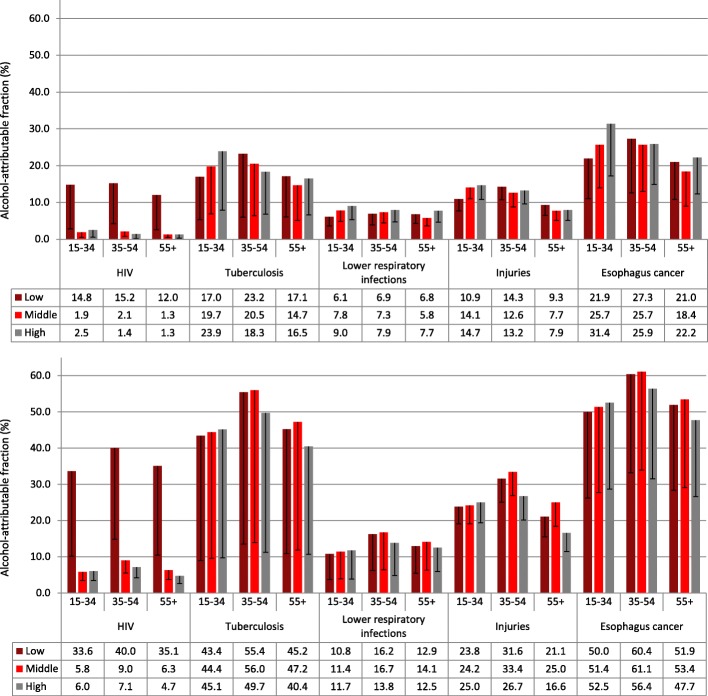
Alcohol-attributable fractions (AAFs) among women (top) and men (bottom) by socioeconomic status (SES), age, and cause of death. Estimates for South Africa in 2015

AAFs for major causes of death are shown in Fig. 2, for details see Additional file 1: Tables S3 and S4. Among men, socioeconomic differences in AAFs were pronounced. Particularly among the middle and older age group (35+), men of middle and low SES had higher AAFs for outcomes such as HIV/AIDS, tuberculosis, injuries, liver cirrhosis, or pancreatitis. For other outcomes, such as cardiovascular diseases, the AAFs were very similar between SES groups. Across SES groups, middle-aged men (35–54) had the highest AAFs. Among women, the socioeconomic pattern was more complex and differences were more pronounced in the youngest age group (15–34). In the latter, women of high SES showed elevated AAFs for some outcomes such as liver cirrhosis, epilepsy, hemorrhagic stroke, and some cancers. However, middle-aged women (35–54) of low SES showed higher AAFs for HIV/AIDS, ischemic stroke, tuberculosis, and pancreatitis.

### Alcohol-attributable mortality

**Fig. 3:**
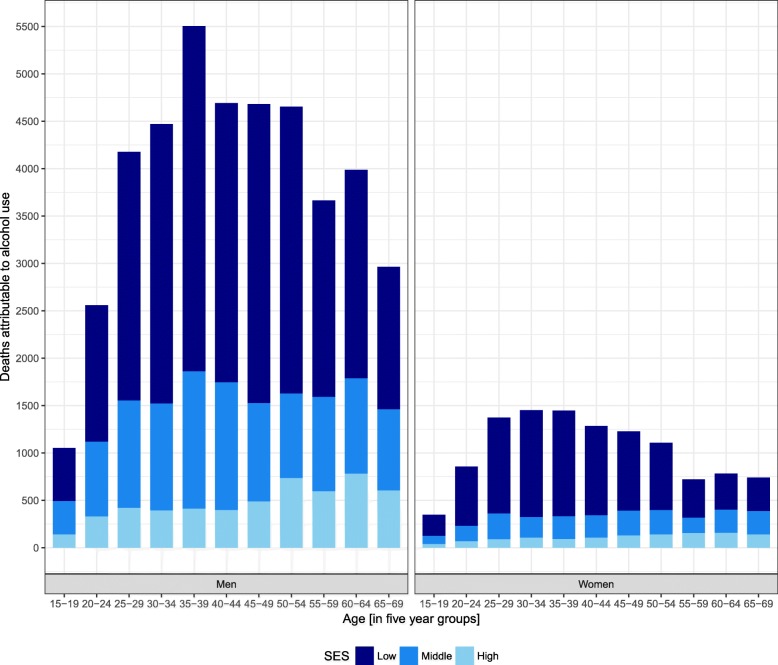
Stacked age distribution of all alcohol-attributable deaths attributable by socioeconomic status (SES) and sex. Estimates for South Africa in 2015. Age was truncated at 70 years

Overall, approximately 62,300 (95% UI 27,000–103,000) adults died from alcohol-attributable causes of death in South Africa in 2015 (Table 1). With a total of approximately 529,400 deaths from all causes in 2015 [40], roughly one in ten deaths was attributable to alcohol use. Roughly 60% of all alcohol-attributable deaths (~39,300 deaths, 95% UI 13,800–71,200) occurred in the low SES group. About a quarter (~17,000 deaths, 95% UI 6600–31,900) occurred in the middle SES group, and roughly 15% (~9000 deaths, 95% UI 3100–17,000) in the high SES group (Table 1). In the high SES group, deaths due to chronic diseases constituted the largest subcategory of alcohol-attributable deaths (~4200 deaths, 95% UI 900–8800). In the middle SES group, chronic (~6200 deaths, 95% UI 1600–13,100) and infectious diseases (~5900 deaths, 95% UI 1900–11,700) contributed approximately the same number of deaths. In the low SES group, the majority of the alcohol-attributable deaths (~24,800 deaths, 95% UI 7000–46,700) occurred due to infectious diseases. The age distribution of all alcohol-attributable deaths by SES and sex is shown in Fig. 3.

**Fig. 4:**
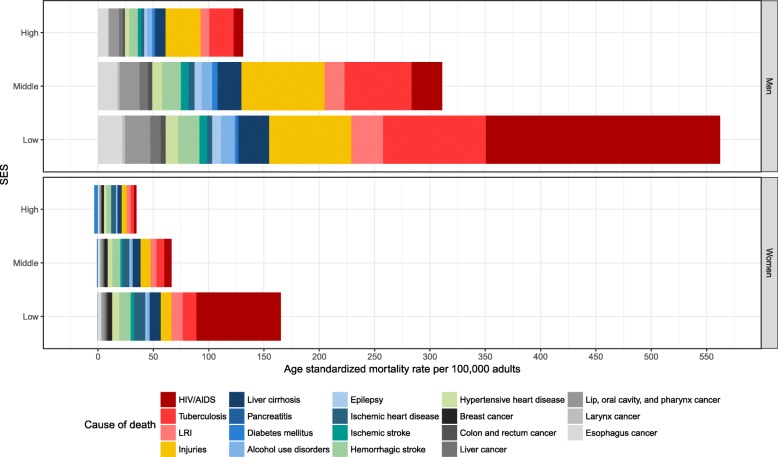
Age-standardized mortality rates per 100,000 attributable to alcohol consumption. Estimates for South Africa in 2015 by socioeconomic status (SES), cause of death, and sex. *LRI *lower respiratory infections

Cause-specific, age-standardized rates of alcohol-attributable mortality (deaths per 100,000 adults) are shown in Fig. 4 (for complete results see Additional file 1: Table S5). Overall, the age-standardized mortality rate from all alcohol-attributable deaths was 727 (95% UI 354–1208) deaths per 100,000 adults in the low, 377 (95% UI 165–687) deaths per 100,000 adults in the middle, and 163 (95% UI 71–289) deaths per 100,000 adults in the high SES group. Thus, persons of low SES had an approximately 4.5-fold alcohol-attributable mortality rate compared to persons of high SES. For persons of middle SES, the alcohol-attributable mortality rate was 1.9 times higher than for persons of high SES. This compares to HRs of all-cause mortality of 2.73 (95% CI 2.30–3.24) for low compared to high SES and 2.07 (95% CI 1.74–2.46) for middle compared to high SES (Additional file 1: Table S2).

### Sensitivity analysis

The complete analysis was repeated using HRs from the DSA data that were based on an asset score that was not projected onto the national distribution. Overall, the sensitivity analysis confirmed the findings of the main analysis. However, as could be expected, the socioeconomic differences were less pronounced, with a total of approximately 12,500 deaths (95% UI 4400–20,900) in the high, 17,200 deaths (95% UI 8500–27,200) in the middle, and 32,600 deaths (95% UI 13100–55,000) in the low SES group. This corresponded to approximately 20%, 28%, and 52% of all alcohol-attributable deaths occurring in the high, middle, and low SES groups, respectively. Detailed results of the sensitivity analyses are shown in Additional file 1: Table S6 and Figures S2 and S3.

## Discussion

This study was the first to quantify alcohol-attributable mortality in South Africa by SES, thereby adding an important dimension to previous analyses of mortality and health burden associated with alcohol use [14, 41]. Furthermore, this study included deaths from HIV/AIDS, which has not been taken into account in previous analyses.

As expected, a lower SES was associated with a clearly elevated mortality rate from alcohol-attributable causes of death. Given the distribution of the race groups across socioeconomic strata, the elevated mortality burden in the low and middle SES groups was largely experienced by black African as well as other non-white population groups.

For deaths from infectious diseases such as HIV/AIDS, tuberculosis, and lower respiratory infections, the socioeconomic differences in the alcohol-attributable mortality rates were particularly wide. These findings are in line with the results of a recent review and meta-analysis investigating the associations between alcohol-attributable morbidity and mortality and SES [42]. Further, a recent study from South Africa also found a higher contribution of alcohol use to socioeconomic differences for self-reported diagnoses of tuberculosis and a relatively lower contribution to diagnoses of diabetes, stroke, or cancer [43].

AAFs were overall elevated for people of low and middle SES, particularly among men. The socioeconomic differences were much less pronounced (and even inverted among women) in the youngest age group (15–34 years). This reflected more similar drinking patterns between persons of low, middle, and high SES in younger ages. Overall, the elevated AAFs for persons of low and middle SES arose from the higher prevalence of binge drinking and higher levels of alcohol use as compared to the prevalence of current drinking per se. The latter was consistently higher among people of high SES. The findings regarding the SES distribution of drinking patterns were in line with other recent evidence from South Africa [9].

Previous research using individual data on SES, exposure to alcohol use, and cause-specific morbidity or mortality showed that persons of lower SES carry a higher risk for alcohol-attributable harm even after adjusting for patterns of alcohol use [44] as well as other risk behavior [45]. The phenomenon that persons of lower SES often carry a higher health burden despite lower levels of alcohol consumption [46–49] has become known as the ‘alcohol harm paradox’ [50]. As SES-specific risk functions were not taken into account in the present study (with the exception of HIV/AIDS for which interaction effects between SES and alcohol use were accounted for [25, 26]), the reported estimates can, overall, be seen as conservative with respect to the socioeconomic differences in AAFs.

The estimate of about one in ten deaths being attributable to alcohol in South Africa in 2015 was higher than the estimate from a previous analysis from South Africa (~7% of all deaths in 2000) [14] as well as the current estimate of the Global Burden of Disease study (~6% of all deaths in 2015) [41]. This can be explained by the inclusion of additional causes of death, most prominently HIV/AIDS, as well as the use of a mortality envelope (i.e., deaths from all risk factors have to add up to the total deaths observed) in the Global Burden of Disease study.

### Strengths and limitations

This study applied a rigorous methodology using the most up to date data available to estimate nationally representative alcohol-attributable mortality rates for all major causes of death known to be causally attributable to alcohol use by age, sex, and SES.

A main limitation of the study relates to the assessment of alcohol exposure in each sociodemographic subgroup. Even though binge drinking and heavy alcohol use are known to be highly prevalent in South Africa [51–53], estimates of over 60 g of pure alcohol per day, on average, are very high. The high levels of consumption among current drinkers could have resulted from a low coverage of alcohol use that has been observed for all major, nationally representative surveys in South Africa in recent years [36]. The triangulation technique, used to estimate the ‘true’ exposure to alcohol, relied on nationally representative estimates of the prevalence of alcohol use in each subgroup and relative levels of alcohol use between subgroups [37]. Consequently, a underestimation of the prevalence of current alcohol use based on the survey data could have led to an overestimation of the levels of alcohol use among current drinkers.

Underreporting and denial of alcohol use due to stigma or memory bias, a high prevalence of heavy and irregular drinking patterns, systematic non-observation of heavy alcohol use due to the sampling frame, and selective non-response were identified as potential causes of the low coverage [54–58]. Recent research estimated that 93% of the alcohol used in Pretoria, South Africa, was consumed in heavy drinking occasions, a drinking pattern that likely contributed to the low coverage [59]. The latter study also used a much more elaborate assessment of alcohol use, which led to considerably higher levels of consumption among current drinkers compared to the assessment in large nationally representative surveys [53].

As a consequence of the low coverage, AAFs could have been overestimated for some causes of death such as cardiovascular diseases [60], liver cirrhosis [61], or pancreatitis [62], with risk functions that are sensitive to high levels of consumption. At the same time, the potential underestimation of the prevalence of current alcohol use could have led to an underestimation of AAFs for causes of death with flatter risk curves and an elevated risk at low levels of consumption such as cancers [63] or lower respiratory infections [64].

As nationally representative cause of death data including reliable information on the SES of the deceased are not available, HRs were used to split the deaths into the three SES groups. Using the projected asset score relied on the assumption that the population of the DSA was representative of the rural and periurban population in Kwazulu-Natal as assessed in the nationally representative survey.

The HRs used for splitting deaths by SES were not age and sex specific, but rather adjusted for age and sex. However, previous studies did not find systematic sex differences in the socioeconomic gradients of cause-specific mortality that could have led to an over- or underestimation of deaths in a specific sociodemographic group [65].

Uncertainty of all estimates was estimated using a Monte Carlo approach. While this is in line with current standards, the intervals depend on the assumed distributions. An alternative approach for future research could be a systematic analysis of the variation in the resulting point estimates when using plausible extreme values of the lowest level parameters similar to a Latin Hypercube approach [66].

### Implications

There are a series of effective policy measures to prevent alcohol-attributable harm such as limitations of availability and affordability of alcohol, restrictions of alcohol-marketing, and improvement of the healthcare system [67–69]. However, such broad brushed measures might fail to address the causes of death found to be most relevant as well as the high risk groups identified in the current study.

When considering alcohol policies in South Africa, it should be taken into account that approximately 23% of the total alcohol consumed in South Africa in 2015 was unrecorded [70]. It is likely that the lion’s share of unrecorded alcohol is consumed by people of lower SES [71]. This means that policies and interventions targeting the consumption of unrecorded alcohol might be more suitable for reducing alcohol-attributable harm in people of low SES than the national alcohol policies listed above. Furthermore, policies could address drinking venues frequented by people of low SES, such as unlicensed alcohol outlets, also called *shebeens* [72]. Even when selling recorded alcohol, *shebeens* often operate outside the legal market, and policy measures to restrict availability and hours of sales do not reach unlicensed *shebeens* or their customers.

There have been attempts to integrate *shebeens* into the legal market; however, the owners are often not able to afford license fees and related taxes or apply the required changes to adhere to the guidelines or they are situated in areas not zoned for business use [73, 74]. The current political strategy seems to focus on police raids, confiscations of liquor, and closing down of unlicensed outlets [73, 74]. However, this practice fails to acknowledge the economic and social importance *shebeens* have for owners and customers [72]. Drivdal and Lawhon [74] proposed a plural regulation of *shebeens* based on a concerted effort of community leaders, *shebeen* owners, and residents, which could be the first step towards enforcement of closing hours, prevention of sales to minors and intoxicated people, and reduction of violence in and around *shebeens* [75].

Targeted interventions on HIV transmissions under the influence of alcohol are another approach to prevent alcohol-attributable mortality and related socioeconomic differences. Alcohol-related HIV risk-reduction interventions, targeted at drinkers in under-resourced areas, have been shown to be effective in reducing unprotected intercourse under the influence of alcohol [76]. Alternatively, HIV/AIDS risk reduction counseling and brief interventions could be targeted at drinkers in sexually transmitted infections clinics and more broadly in primary healthcare clinics and trauma units [77].

Apart from interventions that address alcohol use and drinking environments of people of low SES, socioeconomic differences can be addressed on a structural level [4]. Healthy communities require living environments with affordable housing, clean water, sanitation and electricity, infrastructure and public transit, access to education and healthcare, and safe opportunities to spend leisure time [78, 79]. Marmot et al. suggested that “*health and health equity might not be the aim of all social and economic policies, but they will be a fundamental result*” ([79], p. 1661). Therefore, all policies should be evaluated with respect to their potential effects on health and its respective inequalities.

## Conclusion

This study corroborated the notion that there is substantial heterogeneity in alcohol-attributable mortality by SES. The findings of this, as well as similar studies, can inform resource allocation for preventive measures and interventions in order to target causes of death with the highest disparities and persons that are at highest risk of a premature death. Addressing alcohol use and related risk behavior in low-income areas is a feasible approach to reducing socioeconomic differences in mortality.

## Additional file

Additional file 1: Supplementary information and findings. (PDF 816 kb)

## Abbreviations

AAFs: Alcohol-attributable fractions
CI: Confidence interval
DSA: Demographic Surveillance Area
HR: Hazard ratio
NIDS: National Income Dynamics Study
SES: Socioeconomic status
UI: Uncertainty interval
WHO: World Health Organization
