# Supplementary material for: The socioeconomic profile of alcohol-attributable mortality in South Africa: a modelling study
Source: BMC Med. 2018 Jun 25;16:97. doi: 10.1186/s12916-018-1080-0 (PMC6016129; doi:10.1186/s12916-018-1080-0)
Supplement: Supplementary file 1 — Supplementary information and findings. (PDF 816 kb) [file 12916_2018_1080_MOESM1_ESM.pdf]

# Additional file 1

Causes of death ........ 2

Calculation of asset scores ........ 4

National Income Dynamics Study ........ 4

Demographic Surveillance Area ........ 6

Hazard ratios ........ 8

Alcohol attributable fractions (AAF) ........ 10

Age-standardized mortality rates for all causes of death ........ 16

Sensitivity analysis ........ 20

References ........ 26

## Causes of death

**Table S1** Overview over causes of death used in the comparative risk assessment along with their ICD-10 codes

| Cause of death | ICD 10 codes |
|---|---|
| Communicable diseases | |
| HIV/AIDS | B20-B24.9 |
| Tuberculosis | A10-A14, A15-A19.9, B90-B90.9, K67.3, K93.0, M49.0, P37.0 |
| Lower respiratory infections | A48.1, A70, J09-J15.8, J16-J16.9, J20-J21.9, P23.0-P23.4 |
| Injuries | |
| Injuries | V00-V86.99, V87.2-V87.3, V88.2-V88.3, V90-V98.8, W00-W46.2, W49-W62.9, W64-W70.9, W73-W75.9, W77-W81.9, W83-W94.9, W97.9, W99-X06.9, X08-X39.9, X46-X47, X47.1-X47.8, X48-X48.9, X50-X54.9, X57-X58.9, X60-Y08.9, Y35-Y84.9, Y87.0-Y87.1, Y88-Y88.3, Y89.0-Y89.1 |
| Non-communicable diseases | |
| Liver cirrhosis [a] | B18-B18.9, I85-I85.9, I98.2, K70-K70.9, K71.3-K71.51, K71.7, K72.1-K74.69, K74.9, K75.8-K76.0, K76.6-K76.7, K76.9 |
| Pancreatitis [a] | K85-K86.9 |
| Diabetes mellitus | E10-E10.11, E10.3-E11.1, E11.3-E12.1, E12.3-E13.11, E13.3-E14.1, E14.3-E14.9, P70.0-P70.2, R73-R73.9 |
| Alcohol use disorders [a] | F10-F10.99, G31.2, G72.1, P04.3, Q86.0, R78.0, X45-X45.9 |
| Epilepsy | G40-G41.9 |
| Non-communicable diseases: cardiovascular diseases | |
| Ischemic heart disease | I20-I25.9 |
| Ischemic stroke [b] | G45-G46.8, I63-I63.9, I65-I66.9, I67.2-I67.3, I67.5-I67.6, I69.3-I69.398 |
| Hemorrhagic stroke [b] | I60-I61.9, I62.0-I62.03, I67.0-I67.1, I68.1-I68.2, I69.0-I69.298 |
| Hypertensive heart disease [a] | I11-I11.9 |
| Non-communicable diseases: cancer | |
| Breast cancer [c] | C50-C50.929, D05-D05.92, D24-D24.9, D48.6-D48.62, D49.3, N60-N60.99 |
| Colon and rectum cancer [c] | C18-C21.9, D01.0-D01.3, D12-D12.9, D37.3-D37.5 |

| Cause of death | ICD 10 codes |
|---|---|
| Larynx cancer [c] | C32-C32.9, D02.0, D14.1, D38.0 |
| Liver cancer [c] | C22-C22.9, D13.4 |
| Esophagus cancer [c] | C15-C15.9, D00.1, D13.0 |
| Lip and oral cavity cancer [c]; pharynx cancer [c] | C0-C08.9, D00.00-D00.07, D10.0-D10.5, D11-D11.9, D37.01-D37.04, D37.09; C11-C11.9, D00.08, D10.6, D37.05, C09-C10.9, C12-C13.9, D10.7 |

a The hazard ratio used for splitting the deaths by SES was based on deaths from all causes.
b The hazard ratio used for splitting the deaths by SES was based on deaths from any type of stroke.
c The hazard ratio used for splitting the deaths by SES was based on deaths from any type of cancer.

## Individual secondary data

### NIDS

The National Income Dynamics Study (NIDS, [31]), Wave 4, was conducted from October 2014 to August 2015 by the University of Cape Town. NIDS is a household survey based on a clustered two-stage sampling frame, stratified by district councils (overall response rate 86.5%). While workers' hostels, convents and monasteries were included by the sampling frame, students' hostels, old age homes, hospitals, prisons, and military barracks, as well as homeless people, were excluded. Face-to-face interviews were conducted with all household members. After removing 4,078 observations with missing data on age, sex, SES, alcohol use, or survey weights, the sample comprised 22,741 adults (15+) with complete data. All analyses were performed taking survey weights into account.

It should be noted that in the nationally representative NIDS data, the race groups were highly unequally distributed across the three levels of SES. The vast majority of the white population (84%) were in the high and 13% in the middle SES group while the low SES group was mainly made up by the black African population (93% of the low SES group), followed by the 'coloured' population (6%). The terms 'white', 'black', and 'coloured', originate from the apartheid era. They are used as demographic markers, referring to people of European, African and mixed (African, European and/or Asian) ancestry, respectively. These markers were chosen for their historical significance and because they are commonly used in South Africa nowadays for purposes of sociodemographic monitoring.

## Calculation of asset scores

SES is a multifaceted concept comprising aspects of income, wealth, and assets, occupation and employment, living conditions and education [18-20]. The traditional measures of SES (i.e., occupation, education and income) have downfalls regarding validity over time, disclosure (in the case of income), and gender bias (in the case of occupation) [20].

Measures of SES related to housing conditions, household assets (such as ownership of a refrigerator, TV, car), and access to basic services (i.e., water, sanitation and electricity) have gained importance in the context of low and middle income countries [19, 21, 22]. The assessment of the underlying variables has been found to be straightforward and moderately reliable [23, 24]. Another advantage of asset scores is that they capture multiple facets of SES, such as aspects of living circumstances and sanitation, as well as the ownership of assets that facilitate school attendance, pursuing a profession, accessing health services and other factors that are relevant to one's health [19, 22].

### National Income Dynamics Study

For each household 27 dichotomous variables were available including electricity access of the household and ownership of the following assets: camera, cell phone, computer, donkey cart or ox cart, DVD or Bluray player, electric stove, fridge/freezer, gas stove, grinding mill, HiFi stereo , CD player, MP player, lounge suite, motor boat, motorcycle/scooter, non-motorised boat, paraffin stove, satellite dish, sewing/knitting machine, washing machine, bicycle, microwave, motor vehicle (including bakkie or truck) in running condition, plough, television, tractor, wheelbarrow, and radio.

Additionally the following two categorical variables were available:

- Water access of the household, categorized into 'piped (tab) water in the dwelling', 'Piped (tap) water on site or in yard', 'public tap', 'water-carrier/tanker', 'borehole off site/communal', 'rain-water tank on site', 'well', 'spring', 'borehole on site', 'flowing water/stream', 'dam/pool/stagnant water'.
- The type of toilet available to the household categorized into 'flush toilet with onsite or offsite disposal', 'pit latrine with ventilation pipe', 'pit latrine without ventilation pipe', 'chemical toilet', 'bucket toilet', 'none'.

The variables were combined to a continuous asset score using multiple correspondence analysis (MCA). A two-step selection process was applied for variable selection. First, all variables where over 95% of the participants reported the same category were excluded to assure stability of the model [first selection]. In the first selection ownership of a motor-cycle, non-motorised boat, a motor boat, a cart, a plough, a grinding mill and a tractor were excluded. Then missing values in individual asset variables were imputed based on an iterative MCA algorithm, using the imputeMCA command from the missMCA package [1]. After fitting the MCA on the imputed data, variables for which all categories contributed less than 1.5% to the first dimension were excluded [second selection]. In the second selection ownership of a paraffin stove and a wheelbarrow were excluded. The first dimension resulting from the MCA (explaining 69% of the total variation) was split into tertiles to represent low, middle, and high SES.

### Demographic Surveillance Area

For each household 28 dichotomous variables were available including electricity access of the household and ownership of the following assets: Bed net, bed, bicycle, block maker, car, car battery, cattle, electric cooker with oven, electric hotplate, electric kettle, fridge, gas cooker, hoe/spade/fork, kombi/lorry/tractor, kitchen sink, motorcycle, other life stock, primus cooker, radio, sofa, sewing machine, table and chairs, landline telephone, cell phone, TV, videorecorder, and wheelbarrow. Additionally the following three categorical variables were available:

- Water access of the household, categorized into 'piped water in the dwelling', 'piped water in the yard, water carrier or water tanker', 'piped public tab/kiosk', 'protected spring/rainwater/borehole/well/ flowing river or stream/stagnant water or dam '.
- The type of toilet available to the household categorized into 'flush toilet', 'pit latrine/chemical toilet/ bucket toilet', and 'none'.
- The kind of cooking fuel available to the household categorized into 'electricity from the grid or solar energy', 'electricity from a generator/gas/coal', and 'paraffin/wood'.

The variables referring to car battery, motorcycle, kombi/lorry/tractor, and bed net ownership were excluded in the first selection. The variables referring hoe/spade/fork and block maker ownership were excluded in the second selection. The final MCA used 25 asset variables. The first dimension of the MCA (explaining 57% of the total variation) was split in three equal tertiles for the sensitivity analysis and was mapped onto the national distribution for the main analysis.

## Hazard ratios

The HRs were derived from DSA data using Cox proportional hazards survival analyses for specific causes of death, as well as broader cause-categories, adjusting for age at baseline and sex. When a cause-specific HR was not available or when less than 50 deaths had been observed from a specific cause of death, a broader category (e.g. all strokes or all cancers) or an HR for all-cause mortality was used.

**Table S2** Hazard ratios used for splitting deaths by socioeconomic status. The asset score measuring socioeconomic status was once mapped onto the national asset distribution, once the unmapped original hazard ratios are show

| | Mapped | | | | Unmapped | | | |
|---|---|---|---|---|---|---|---|---|
| **Cause of death** | **HR** | **95% CI** | **N** | **Deaths** | **HR** | | **N** | **Deaths** |
| All causes | 1 | | 5,222 | 133 | 1 | | 29,012 | 1,710 |
| | 2.07 | (1.74-2.46) | 33,942 | 2,889 | 1.48 | (1.40-1.57) | 29,007 | 4,069 |
| | 2.73 | (2.30-3.24) | 47,806 | 8,479 | 1.62 | (1.53-1.71) | 28,951 | 5,722 |
| HIV/AIDS | 1 | | 5,222 | 17 | 1 | | 29,012 | 256 |
| | 2.69 | (1.65-4.36) | 33,942 | 411 | 1.60 | (1.38-1.86) | 29,007 | 574 |
| | 4.24 | (2.62-6.85) | 47,806 | 1,249 | 2.04 | (1.77-2.35) | 28,951 | 847 |
| Tuberculosis | 1 | | 5,222 | 44 | 1 | | 29,012 | 566 |
| | 2.42 | (1.79-3.28) | 33,942 | 997 | 1.88 | (1.71-2.07) | 29,007 | 1,530 |
| | 3.84 | (2.85-5.17) | 47,806 | 3,093 | 2.14 | (1.95-2.35) | 28,951 | 2,038 |
| Pneumonia | 1 | | 5,222 | 2 | 1 | | 29,012 | 28 |
| | 1.98 | (0.48-8.18) | 33,942 | 46 | 1.50 | (0.97-2.32) | 29,007 | 73 |
| | 3.45 | (0.85-13.93) | 47,806 | 195 | 2.12 | (1.41-3.20) | 28,951 | 142 |
| Injuries | 1 | | 5,222 | 18 | 1 | | 29,012 | 215 |
| | 2.02 | (1.26-3.25) | 33,942 | 335 | 1.26 | (1.07-1.49) | 29,007 | 389 |
| | 2.15 | (1.34-3.43) | 47,806 | 662 | 1.22 | (1.03-1.44) | 28,951 | 411 |
| Diabetes mellitus | 1 | | 5,222 | 3 | 1 | | 29,012 | 49 |
| | 1.42 | (0.45-4.52) | 33,942 | 77 | 0.74 | (0.52-1.06) | 29,007 | 84 |
| | 1.08 | (0.34-3.40) | 47,806 | 185 | 0.72 | (0.51-1.00) | 28,951 | 132 |
| Ischemic heart disease | 1 | | 5,222 | 1 | 1 | | 29,012 | 10 |
| | 1.52 | (0.20-11.40) | 33,942 | 19 | 1.50 | (0.72-3.11) | 29,007 | 26 |
| | 2.09 | (0.29-15.17) | 47,806 | 63 | 1.96 | (0.98-3.92) | 28,951 | 47 |
| Stroke | 1 | | 5,222 | 3 | 1 | | 29,012 | 52 |
| | 1.94 | (0.62-6.13) | 33,942 | 105 | 1.55 | (1.14-2.11) | 29,007 | 184 |
| | 2.38 | (0.76-7.44) | 47,806 | 410 | 1.44 | (1.07-1.95) | 28,951 | 282 |

| Cause of death | Mapped | | | | Unmapped | | | |
|---|---|---|---|---|---|---|---|---|
| | **HR** | **95% CI** | **N** | **Deaths** | **HR** | | **N** | **Deaths** |
| All cancer | 1 | | 5,222 | 9 | 1 | | 29,012 | 109 |
| | 1.75 | (0.90-3.42) | 33,942 | 200 | 1.46 | (1.17-1.83) | 29,007 | 293 |
| | 2.26 | (1.17-4.37) | 47,806 | 664 | 1.65 | (1.33-2.04) | 28,951 | 471 |

HR Hazard ratio; CI confidence interval

## Alcohol attributable fractions (AAF)

**Table S3** Alcohol-attributable fractions (AAF) among men by cause of death, socioeconomic status (SES) and age-group in South Africa, 2015

| SES | | Low | | Middle | | High | |
|---|---|---|---|---|---|---|---|
| **Cause of death** | **Age** | **AAF** | **95% UI** | **AAF** | **95% UI** | **AAF** | **95% UI** |
| HIV/AIDS | 15-34 | 33.6 | (10.1-57.4) | 5.8 | (3.4-8.6) | 6.0 | (3.4-8.8) |
| | 35-54 | 40.0 | (14.8-63.1) | 9.0 | (5.5-12.9) | 7.1 | (4.2-10.4) |
| | 55+ | 35.1 | (10.5-59.2) | 6.3 | (3.7-9.4) | 4.7 | (2.6-7.2) |
| Tuberculosis | 15-34 | 43.4 | (9.0-78.5) | 44.4 | (9.6-78.9) | 45.1 | (9.7-79.2) |
| | 35-54 | 55.4 | (13.5-86.2) | 56.0 | (13.9-86.5) | 49.7 | (11.2-82.2) |
| | 55+ | 45.2 | (10.9-79.4) | 47.2 | (11.9-80.8) | 40.4 | (10.7-74.3) |
| Lower respiratory infections | 15-34 | 10.8 | (3.7-19.1) | 11.4 | (3.9-20.0) | 11.7 | (3.8-20.7) |
| | 35-54 | 16.2 | (6.2-27.2) | 16.7 | (6.4-27.7) | 13.8 | (4.8-23.6) |
| | 55+ | 12.9 | (5.4-21.4) | 14.1 | (6.3-23.0) | 12.5 | (5.9-19.8) |
| Injuries | 15-34 | 23.8 | (19.1-28.9) | 24.2 | (19.1-29.5) | 25.0 | (19.4-30.8) |
| | 35-54 | 31.6 | (25.0-38.1) | 33.4 | (26.9-39.8) | 26.7 | (20.1-33.4) |
| | 55+ | 21.1 | (15.5-26.7) | 25.0 | (18.4-31.8) | 16.6 | (11.4-21.9) |
| Liver Cirrhosis | 15-34 | 69.8 | (59.4-78.2) | 70.4 | (60.0-78.7) | 70.7 | (60.2-79.0) |
| | 35-54 | 79.7 | (71.6-85.9) | 80.1 | (72.1-86.3) | 74.7 | (65.1-82.2) |
| | 55+ | 71.0 | (60.5-79.3) | 72.8 | (62.5-81.0) | 65.5 | (53.4-75.3) |
| Pancreatitis | 15-34 | 41.7 | (21.9-62.0) | 42.7 | (22.7-63.1) | 43.4 | (23.4-63.6) |
| | 35-54 | 53.5 | (30.8-73.2) | 54.2 | (31.2-73.6) | 47.9 | (26.7-67.7) |
| | 55+ | 43.5 | (23.9-63.6) | 45.5 | (25.4-65.6) | 38.8 | (21.3-58.3) |
| Diabetes mellitus | 15-34 | 0.4 | (-8.5-10.9) | 0.2 | (-9.4-11.2) | -0.3 | (-10.7-11.2) |

| SES | | Low | | Middle | | High | |
|---|---|---|---|---|---|---|---|
| Cause of death | Age | AAF | 95% UI | AAF | 95% UI | AAF | 95% UI |
| | 35-54 | 2.9 | (-10.6-17.3) | 3.0 | (-11.0-17.7) | 0.7 | (-11.4-13.9) |
| | 55+ | 2.0 | (-8.9-13.6) | 3.1 | (-9.0-15.6) | 2.6 | (-8.3-14.0) |
| Epilepsy | 15-34 | 28.4 | (21.1-36.1) | 29.4 | (21.8-37.2) | 30.1 | (22.4-38.1) |
| | 35-54 | 38.5 | (29.9-47.2) | 39.2 | (30.5-47.9) | 34.0 | (25.7-42.4) |
| | 55+ | 30.5 | (22.9-38.4) | 32.2 | (24.2-40.4) | 27.3 | (20.3-34.9) |
| Ischemic heart disease | 15-34 | -1.3 | (-11.2-5.1) | -1.9 | (-11.2-5.1) | -2.8 | (-11.2-5.1) |
| | 35-54 | 1.7 | (-7.4-4.3) | 1.9 | (-7.4-4.3) | -1.5 | (-7.4-4.3) |
| | 55+ | 1.8 | (-3.2-8.6) | 3.0 | (-3.3-8.6) | 2.4 | (-3.3-8.7) |
| Ischemic stroke | 15-34 | 6.3 | (-0.1-17.0) | 6.4 | (-0.3-16.9) | 5.8 | (0.0-17.0) |
| | 35-54 | 12.2 | (1.0-17.1) | 12.5 | (0.8-17.0) | 8.1 | (1.0-17.1) |
| | 55+ | 8.1 | (-1.5-21.3) | 9.8 | (-1.4-21.4) | 8.6 | (-1.6-21.1) |
| Hemorrhagic stroke | 15-34 | 16.2 | (10.3-22.5) | 16.9 | (10.8-23.6) | 17.3 | (11.2-23.9) |
| | 35-54 | 23.6 | (15.0-32.4) | 24.2 | (15.4-33.2) | 20.2 | (13.0-28.0) |
| | 55+ | 19.0 | (10.9-27.8) | 20.7 | (11.5-30.6) | 18.3 | (9.0-28.5) |
| Hypertensive heart disease | 15-34 | 17.6 | (14.2-21.0) | 18.6 | (15.1-22.2) | 19.5 | (15.7-23.3) |
| | 35-54 | 23.6 | (19.7-27.5) | 24.2 | (20.2-28.1) | 21.9 | (17.8-26.0) |
| | 55+ | 19.8 | (16.0-23.6) | 20.9 | (16.8-25.0) | 18.9 | (14.9-22.9) |
| Colon and rectum cancer | 15-34 | 14.0 | (9.9-18.4) | 14.7 | (10.3-19.3) | 15.1 | (10.6-19.9) |
| | 35-54 | 20.4 | (14.9-26.1) | 20.9 | (15.3-26.7) | 17.6 | (12.5-22.9) |
| | 55+ | 16.1 | (11.6-20.8) | 17.4 | (12.6-22.5) | 15.1 | (10.9-19.6) |
| Liver cancer | 15-34 | 45.2 | (34.5-54.4) | 45.4 | (34.4-54.9) | 45.3 | (33.6-55.2) |
| | 35-54 | 60.2 | (51.1-67.8) | 60.8 | (51.6-68.4) | 51.2 | (40.0-60.6) |
| | 55+ | 46.7 | (35.8-56.1) | 49.6 | (38.8-59.0) | 39.6 | (28.3-49.9) |

| SES | | Low | | Middle | | High | |
|---|---|---|---|---|---|---|---|
| **Cause of death** | **Age** | **AAF** | **95% UI** | **AAF** | **95% UI** | **AAF** | **95% UI** |
| Lip, oral cavity, and pharynx cancer | 15-34 | 50.7 | (43.3-57.6) | 51.8 | (44.2-58.7) | 52.7 | (44.8-59.7) |
| | 35-54 | 62.2 | (55.2-68.4) | 62.8 | (55.8-69.0) | 57.0 | (49.3-63.9) |
| | 55+ | 52.5 | (44.7-59.5) | 54.3 | (46.1-61.7) | 47.5 | (39.0-55.3) |
| Larynx cancer | 15-34 | 29.4 | (22.6-36.3) | 30.4 | (23.4-37.5) | 31.3 | (24.1-38.5) |
| | 35-54 | 39.3 | (31.4-47.0) | 40.0 | (32.1-47.7) | 35.0 | (27.3-42.7) |
| | 55+ | 31.5 | (24.4-38.7) | 33.1 | (25.7-40.7) | 28.4 | (21.6-35.4) |
| Esophagus cancer | 15-34 | 50.0 | (26.2-73.8) | 51.4 | (27.7-74.5) | 52.5 | (28.7-75.0) |
| | 35-54 | 60.4 | (33.2-82.2) | 61.1 | (33.9-82.6) | 56.4 | (31.5-78.5) |
| | 55+ | 51.9 | (28.3-74.9) | 53.4 | (29.1-76.4) | 47.7 | (26.6-70.4) |

SES socioeconomic status; AAF alcohol-attributable fraction; UI uncertainty interval

**Table S4** Alcohol-attributable fractions (AAF) among women by cause of death, socioeconomic status (SES) and age-group in South Africa, 2015

| SES | | Low | | Middle | | High | |
|---|---|---|---|---|---|---|---|
| **Cause of death** | **Age** | **AAF** | **95% UI** | **AAF** | **95% UI** | **AAF** | **95% UI** |
| HIV/AIDS | 15-34 | 14.8 | (2.8-32.6) | 1.9 | (0.5-3.4) | 2.5 | (0.6-4.6) |
| | 35-54 | 15.2 | (4.2-31.6) | 2.1 | (0.8-3.6) | 1.4 | (0.2-3.1) |
| | 55+ | 12.0 | (2.6-27.1) | 1.3 | (0.5-2.3) | 1.3 | (0.3-2.7) |
| Tuberculosis | 15-34 | 17.0 | (5.3-43.5) | 19.7 | (6.9-46.4) | 23.9 | (7.9-53.1) |
| | 35-54 | 23.2 | (6.0-57.1) | 20.5 | (6.4-50.0) | 18.3 | (6.8-40.7) |
| | 55+ | 17.1 | (6.1-43.6) | 14.7 | (5.1-39.2) | 16.5 | (6.6-38.4) |
| Lower respiratory infections | 15-34 | 6.1 | (3.6-9.0) | 7.8 | (4.8-11.4) | 9.0 | (5.3-13.5) |
| | 35-54 | 6.9 | (3.9-10.6) | 7.3 | (4.4-10.9) | 7.9 | (4.7-11.6) |
| | 55+ | 6.8 | (4.3-9.8) | 5.8 | (3.6-8.5) | 7.7 | (4.6-11.2) |
| Injuries | 15-34 | 10.9 | (7.7-14.1) | 14.1 | (11-17.4) | 14.7 | (10.8-18.6) |
| | 35-54 | 14.3 | (10.7-18.1) | 12.6 | (8.8-16.4) | 13.2 | (9.6-17.0) |
| | 55+ | 9.3 | (6.5-12.1) | 7.7 | (5.1-10.7) | 7.9 | (5.1-11.0) |
| Liver Cirrhosis | 15-34 | 59.0 | (44.0-73.1) | 64.4 | (49.5-78) | 69.4 | (55.4-80.8) |
| | 35-54 | 65.0 | (52.1-76.3) | 63.9 | (49.3-76.9) | 64.4 | (49.7-78.0) |
| | 55+ | 59.8 | (43.7-75.3) | 55.7 | (38.9-72.3) | 61.6 | (44.7-77.6) |
| Pancreatitis | 15-34 | 12.4 | (1.1-42.5) | 13.4 | (0.9-44.5) | 15.1 | (-1-50.7) |
| | 35-54 | 20.4 | (3.0-57.4) | 15.9 | (1.9-49.4) | 7.7 | (-2.6-34.4) |
| | 55+ | 14.0 | (3.1-43.2) | 11.7 | (2.4-38.5) | 9.8 | (0.7-34.5) |
| Diabetes mellitus | 15-34 | -1.7 | (-4.5-1.9) | -2.8 | (-6.4-1.8) | -5.2 | (-10.2-1) |
| | 35-54 | -0.5 | (-3.8-3.8) | -1.6 | (-5.0-2.8) | -6.6 | (-10.8--1.4) |

| SES | | Low | | Middle | | High | |
|---|---|---|---|---|---|---|---|
| Cause of death | Age | AAF | 95% UI | AAF | 95% UI | AAF | 95% UI |
| | 55+ | -0.3 | (-3.0-3.1) | -0.4 | (-2.9-2.5) | -2.8 | (-6.5-1.4) |
| Epilepsy | 15-34 | 11.3 | (7.3-15.5) | 13.7 | (8.9-18.7) | 16.6 | (10.6-22.7) |
| | 35-54 | 14.5 | (9.9-19.3) | 13.7 | (8.8-18.5) | 13.4 | (8.7-18.7) |
| | 55+ | 11.6 | (7.8-15.6) | 10.0 | (6.4-13.9) | 12.1 | (7.8-16.9) |
| Ischemic heart disease | 15-34 | 4.2 | (-4.3-17.3) | 5.0 | (-4.2-17.4) | 5.0 | (-4.4-17.4) |
| | 35-54 | 6.0 | (-3.7-11.1) | 5.3 | (-3.8-11.0) | 2.9 | (-3.8-11.1) |
| | 55+ | 4.8 | (-0.3-10.0) | 4.0 | (-0.3-10.0) | 4.4 | (-0.4-10.0) |
| Ischemic stroke | 15-34 | 4.4 | (-8.2-55.1) | 3.6 | (-8.3-55.2) | 2.6 | (-8.3-54.1) |
| | 35-54 | 10.2 | (-12.0-11.0) | 5.7 | (-12.1-10.8) | -3.2 | (-12.4-10.7) |
| | 55+ | 3.4 | (-6.1-15.7) | 2.7 | (-6.4-15.2) | 0.2 | (-5.3-16.0) |
| Hemorrhagic stroke | 15-34 | 11.4 | (3.5-22.5) | 13.3 | (3.9-25.8) | 16.8 | (5.7-31.4) |
| | 35-54 | 15.9 | (5.8-30.2) | 13.9 | (4.4-26.8) | 12.5 | (3.7-24.4) |
| | 55+ | 11.0 | (2.7-22.3) | 9.4 | (2.3-19.8) | 10.7 | (2.1-22.2) |
| Hypertensive heart disease | 15-34 | 8.5 | (4.8-13.3) | 10.4 | (6.0-16.0) | 12.3 | (6.7-19.4) |
| | 35-54 | 10.6 | (6.1-16.4) | 10.3 | (5.9-15.9) | 9.5 | (5.3-15.3) |
| | 55+ | 9.1 | (5.6-13.5) | 7.8 | (4.6-11.9) | 9.4 | (5.4-14.4) |
| Breast cancer | 15-34 | 9.5 | (6.5-12.4) | 11.7 | (8.0-15.2) | 14.1 | (9.5-18.5) |
| | 35-54 | 11.9 | (8.6-14.9) | 11.5 | (7.8-14.9) | 11.6 | (7.8-15.5) |
| | 55+ | 10.0 | (7.0-12.8) | 8.5 | (5.7-11.3) | 10.6 | (7.0-14.3) |
| Colon and rectum cancer | 15-34 | 6.9 | (4.8-9.2) | 8.8 | (6.1-11.6) | 10.3 | (7.1-13.7) |
| | 35-54 | 8.1 | (5.8-10.6) | 8.4 | (5.8-11.0) | 8.8 | (6.0-11.9) |
| | 55+ | 7.6 | (5.3-10.0) | 6.5 | (4.4-8.7) | 8.4 | (5.5-11.6) |
| Liver cancer | 15-34 | 15.1 | (6.4-23.7) | 16.4 | (7.1-26.3) | 18.9 | (7.7-31.1) |

| SES | | Low | | Middle | | High | |
|---|---|---|---|---|---|---|---|
| **Cause of death** | **Age** | **AAF** | **95% UI** | **AAF** | **95% UI** | **AAF** | **95% UI** |
| | 35-54 | 25.3 | (14.6-33.6) | 19.2 | (8.5-28.6) | 11.8 | (5.3-21.7) |
| | 55+ | 16.7 | (8.3-24.3) | 14.1 | (6.6-21.6) | 12.4 | (5.9-21.1) |
| Lip, oral cavity, and pharynx cancer | 15-34 | 21.1 | (12.5-28.2) | 24.2 | (14.4-32.2) | 29.3 | (17.4-38.8) |
| | 35-54 | 28.1 | (19.7-35.0) | 25.1 | (15.3-32.8) | 22.7 | (13.5-31.9) |
| | 55+ | 20.8 | (13.0-27.3) | 18.1 | (10.6-24.9) | 20.2 | (11.9-28.4) |
| Larynx cancer | 15-34 | 11.9 | (7.7-15.9) | 14.4 | (9.4-19.2) | 17.5 | (11.3-23.5) |
| | 35-54 | 14.9 | (10.4-19.4) | 14.2 | (9.3-18.9) | 14.2 | (9.2-19.6) |
| | 55+ | 12.0 | (8.1-15.8) | 10.3 | (6.7-14.1) | 12.7 | (8.2-17.5) |
| Esophagus cancer | 15-34 | 21.9 | (11.0-40.5) | 25.7 | (13.9-44.6) | 31.4 | (17.2-51.5) |
| | 35-54 | 27.3 | (12.6-50.9) | 25.7 | (13.0-46.0) | 25.9 | (14.9-42.3) |
| | 55+ | 21.0 | (10.8-39.2) | 18.4 | (9.0-35.7) | 22.2 | (12.3-38.2) |

SES socioeconomic status; AAF alcohol-attributable fraction; UI uncertainty interval

## Age-standardized mortality rates for all causes of death

**Table S5** Age-standardized mortality rates per 100,000 adult (15+) population for alcohol-attributable causes of death in South Africa in 2015, by SES and sex (main analysis)

| | | Men | | Women | |
|---|---|---|---|---|---|
| **Cause of death** | **SES** | **Mortality rate** | **95% UI** | **Mortality rate** | **95% UI** |
| HIV/AIDS | Low | 328.4 | (111.8-328.4) | 143.2 | (35.2-143.2) |
| | Middle | 43.7 | (15.7-43.7) | 11.0 | (3.0-11.0) |
| | High | 12.8 | (5.3-12.8) | 4.0 | (0.9-4.0) |
| Tuberculosis | Low | 142.0 | (44.2-142.0) | 24.3 | (6.4-24.3) |
| | Middle | 95.9 | (28.4-95.9) | 14.3 | (3.7-14.3) |
| | High | 34.8 | (10.6-34.8) | 5.7 | (1.8-5.7) |
| Lower respiratory infections | Low | 56.3 | (7.7-56.3) | 17.9 | (3.1-17.9) |
| | Middle | 48.5 | (3.4-48.5) | 13.6 | (1.1-13.6) |
| | High | 17.1 | (2.4-17.1) | 7.3 | (1.1-7.3) |
| Injuries | Low | 100.8 | (50.4-100.8) | 13.4 | (6.4-13.4) |
| | Middle | 105.1 | (49.3-105.1) | 12.7 | (5.9-12.7) |
| | High | 42.0 | (22.6-42.0) | 6.2 | (3.3-6.2) |
| Liver cirrhosis | Low | 31.1 | (23.3-31.1) | 11.9 | (7.8-11.9) |
| | Middle | 24.8 | (17.5-24.8) | 8.9 | (5.4-8.9) |
| | High | 10.7 | (7.8-10.7) | 4.5 | (2.9-4.5) |
| Pancreatitis | Low | 1.7 | (0.9-1.7) | 0.4 | (0.1-0.4) |
| | Middle | 1.3 | (0.7-1.3) | 0.3 | (0.0-0.3) |
| | High | 0.6 | (0.3-0.6) | 0.1 | (0.0-0.1) |
| Diabetes mellitus | Low | 16.8 | (-9.1-16.8) | 3.4 | (-3.7-3.4) |

| Cause of death | SES | Men | | Women | |
|---|---|---|---|---|---|
| | | Mortality rate | 95% UI | Mortality rate | 95% UI |
| | Middle | 24.6 | (-11.7-24.6) | 3.2 | (-4.9-3.2) |
| | High | 13.4 | (-7.2-13.4) | 0.5 | (-7.8-0.5) |
| Alcohol use disorders | Low | 12.6 | (11.2-14.0) | 2.5 | (2.3-2.8) |
| | Middle | 9.6 | (8.3-10.9) | 1.9 | (1.7-2.2) |
| | High | 4.6 | (4.2-5.1) | 0.9 | (0.8-1.0) |
| Epilepsy | Low | 9.5 | (6.6-9.5) | 1.7 | (1.1-1.7) |
| | Middle | 7.7 | (5.1-7.7) | 1.3 | (0.8-1.3) |
| | High | 3.2 | (2.2-3.2) | 0.7 | (0.4-0.7) |
| Ischemic heart disease | Low | 27.3 | (-7.8-27.3) | 26.7 | (-0.1-26.7) |
| | Middle | 24.7 | (-6.3-24.7) | 26.0 | (-0.1-26.0) |
| | High | 12.2 | (-3.5-12.2) | 13.7 | (-0.1-13.7) |
| Ischemic stroke | Low | 22.3 | (-1.0-22.3) | 15.8 | (-6.1-15.8) |
| | Middle | 19.8 | (-0.8-19.8) | 13.7 | (-5.6-13.7) |
| | High | 8.7 | (-0.5-8.7) | 6.8 | (-2.2-6.8) |
| Hemorrhagic stroke | Low | 36.5 | (6.5-36.5) | 23.8 | (2.4-23.8) |
| | Middle | 37.2 | (4.9-37.2) | 19.4 | (1.5-19.4) |
| | High | 14.6 | (2.9-14.6) | 9.8 | (0.9-9.8) |
| Hypertensive heart disease | Low | 13.7 | (9.0-13.7) | 9.4 | (4.1-9.4) |
| | Middle | 11.1 | (7.0-11.1) | 6.4 | (2.6-6.4) |
| | High | 4.8 | (3.1-4.8) | 3.6 | (1.4-3.6) |
| Breast cancer | Low | n.a. | | 6.1 | (2.3-6.1) |
| | Middle | n.a. | | 4.8 | (1.5-4.8) |

| Cause of death | SES | Men: Mortality rate | Men: 95% UI | Women: Mortality rate | Women: 95% UI |
|---|---|---|---|---|---|
| | High | n.a. | | 2.8 | (1.2-2.8) |
| Colon and rectum cancer | Low | 6.8 | (2.5-6.8) | 2.0 | (0.7-2.0) |
| | Middle | 6.3 | (1.9-6.3) | 1.5 | (0.5-1.5) |
| | High | 2.7 | (1.1-2.7) | 1.0 | (0.4-1.0) |
| Liver cancer | Low | 13.7 | (5.5-13.7) | 2.3 | (0.7-2.3) |
| | Middle | 12.5 | (4.0-12.5) | 1.7 | (0.4-1.7) |
| | High | 5.1 | (2.2-5.1) | 0.8 | (0.2-0.8) |
| Lip, oral cavity, and pharynx cancer | Low | 32.2 | (13.6-32.2) | 4.1 | (1.5-4.1) |
| | Middle | 29.6 | (9.5-29.6) | 3.2 | (0.9-3.2) |
| | High | 12.8 | (5.9-12.8) | 1.8 | (0.6-1.8) |
| Larynx cancer | Low | 3.7 | (1.5-3.7) | 0.2 | (0.1-0.2) |
| | Middle | 3.4 | (1.0-3.4) | 0.1 | (0.0-0.1) |
| | High | 1.4 | (0.6-1.4) | 0.1 | (0.0-0.1) |
| Esophagus cancer | Low | 35.9 | (11.0-35.9) | 5.8 | (1.5-5.8) |
| | Middle | 31.4 | (7.9-31.4) | 4.4 | (0.9-4.4) |
| | High | 14.4 | (4.8-14.4) | 2.5 | (0.7-2.5) |

SES socioeconomic status; UI uncertainty interval; n.a. not applicable

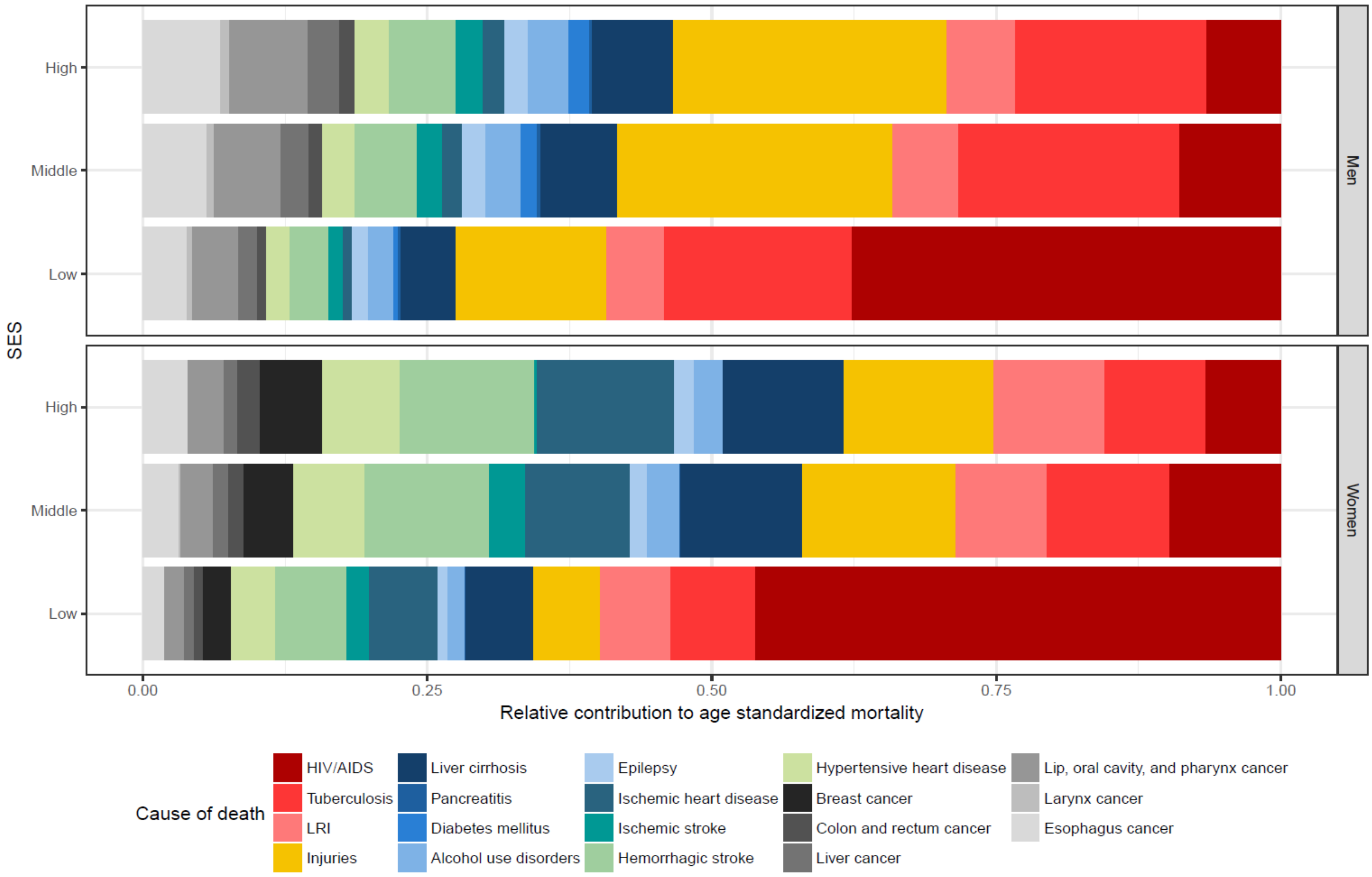


**Figure S1** Relative contribution of causes of death to the overall age standardized mortality rates by SES for men and women in South Africa, 2015

## Sensitivity analysis

As in the main analysis, the category with the most deaths among persons of high SES was alcohol-attributable deaths due to chronic diseases (about 5,300 deaths, 95% UI 1,600-9,900) whereas alcohol-attributable deaths due to infectious diseases constituted the largest sub-group in persons low SES (about 20,400 deaths, 95% UI 6,200-36,400). In the middle SES group deaths due to chronic (6,200, 95% UI 2,600-10,600) and infectious diseases (6,400, 95% UI 2,400-10,800) were roughly equally common. The age-standardized mortality rate from all alcohol-attributable deaths was 603 (95% UI 337-913) deaths per 100,000 adults in the low, 380 (95% UI 219-566) deaths per 100,000 adults in the middle, and 225 (95% UI 123-346) deaths per 100,000 adults in the high SES group. Thereby the alcohol-attributable mortality rate was still about 2.6 times higher at low, and about 1.4 times higher at middle SES compared to high SES. This compares to HRs of all-cause mortality of 1.48 (95% CI 1.40-1.57) for low compared to high SES and 1.62 (95% CI 1.53-1.71) for middle compared to high SES using the un-projected asset score (Additional file 1: Table S2).

**Table S6** Sensitivity analysis: age-standardized mortality rates per 100,000 adult (15+) population for alcohol-attributable causes of death in South Africa in 2015, by SES and sex using unmapped hazard ratios to split deaths

| | | Men | | Women | |
|---|---|---|---|---|---|
| **Cause of death** | **SES** | **Mortality rate** | **95% UI** | **Mortality rate** | **95% UI** |
| HIV/AIDS | Low | 174.0 | (98.4-251.9) | 62.1 | (30.3-111.0) |
| | Middle | 28.2 | (19.7-37.9) | 6.5 | (3.4-9.8) |
| | High | 14.7 | (10.2-19.7) | 3.8 | (1.7-6.3) |

| Cause of death | SES | Men | | Women | |
|---|---|---|---|---|---|
| | | Mortality rate | 95% UI | Mortality rate | 95% UI |
| Tuberculosis | Low | 74.3 | (36.2-110.7) | 9.9 | (5.3-19.2) |
| | Middle | 67.3 | (33.2-99.0) | 8.0 | (4.3-15.3) |
| | High | 31.6 | (15.7-48.3) | 4.5 | (2.6-8.0) |
| Lower respiratory infections | Low | 24.1 | (11.8-39.5) | 8.7 | (5.4-12.8) |
| | Middle | 18.4 | (8.9-31.5) | 5.5 | (3.2-8.8) |
| | High | 10.8 | (5.8-16.9) | 4.7 | (2.9-6.9) |
| Injuries | Low | 62.5 | (51.9-73.9) | 8.1 | (6.5-9.8) |
| | Middle | 69.6 | (57.7-82.4) | 8.3 | (6.7-10.1) |
| | High | 47.0 | (39.1-55.1) | 6.8 | (5.6-8.2) |
| Liver cirrhosis | Low | 22.5 | (20.0-24.7) | 8.1 | (6.6-9.6) |
| | Middle | 21.0 | (18.6-23.0) | 7.1 | (5.6-8.5) |
| | High | 12.9 | (11.2-14.4) | 5.1 | (4.2-6.1) |
| Pancreatitis | Low | 1.0 | (0.7-1.3) | 0.1 | (0.0-0.3) |
| | Middle | 1.0 | (0.7-1.3) | 0.1 | (0.0-0.3) |
| | High | 0.6 | (0.4-0.8) | 0.1 | (0.0-0.2) |
| Diabetes mellitus | Low | 2.1 | (-7.4-12.5) | -0.3 | (-2.9-2.8) |
| | Middle | 3.1 | (-7.7-14.8) | -0.6 | (-3.1-2.2) |
| | High | 3.2 | (-9.8-16.9) | -4.8 | (-9.5-0.7) |
| Alcohol use disorders | Low | 10.3 | (9.9-10.7) | 2.1 | (2.0-2.2) |
| | Middle | 9.4 | (9.0-9.8) | 1.9 | (1.8-2.0) |
| | High | 6.3 | (6.2-6.5) | 1.3 | (1.3-1.3) |
| Epilepsy | Low | 6.6 | (5.6-7.7) | 1.2 | (0.9-1.4) |

| Cause of death | SES | Men | | Women | |
|---|---|---|---|---|---|
| | | **Mortality rate** | **95% UI** | **Mortality rate** | **95% UI** |
| | Middle | 6.3 | (5.3-7.3) | 1.0 | (0.8-1.2) |
| | High | 3.8 | (3.2-4.4) | 0.8 | (0.6-1.0) |
| Ischemic heart disease | Low | 4.3 | (-7.4-20.9) | 9.8 | (-0.2-21.1) |
| | Middle | 5.5 | (-5.9-17.1) | 6.2 | (-0.2-17.7) |
| | High | 2.6 | (-3.8-10.3) | 4.3 | (-0.1-10.6) |
| Ischemic stroke | Low | 5.6 | (-0.9-14.8) | 2.7 | (-4.7-12.0) |
| | Middle | 7.2 | (-0.9-16.0) | 2.2 | (-5.3-12.7) |
| | High | 4.1 | (-0.7-9.9) | 0.1 | (-2.8-8.5) |
| Hemorrhagic stroke | Low | 15.2 | (9.0-22.6) | 8.3 | (2.7-16.4) |
| | Middle | 17.6 | (10.1-26.8) | 7.7 | (2.5-15.7) |
| | High | 10.0 | (5.4-15.3) | 5.5 | (1.5-11.0) |
| Hypertensive heart disease | Low | 9.2 | (7.6-10.8) | 5.4 | (3.5-7.7) |
| | Middle | 8.8 | (7.2-10.5) | 4.2 | (2.7-6.3) |
| | High | 5.4 | (4.4-6.4) | 3.4 | (2.1-5.0) |
| Breast cancer | Low | n.a. | | 3.7 | (2.7-4.6) |
| | Middle | n.a. | | 2.9 | (2.1-3.8) |
| | High | n.a. | | 2.3 | (1.7-3.0) |
| Colon and rectum cancer | Low | 3.9 | (2.8-5.1) | 1.2 | (0.8-1.6) |
| | Middle | 3.7 | (2.7-5.0) | 0.9 | (0.6-1.3) |
| | High | 2.2 | (1.6-2.8) | 0.8 | (0.6-1.1) |
| Liver cancer | Low | 8.3 | (6.5-10.1) | 1.3 | (0.8-1.8) |
| | Middle | 7.7 | (6.0-9.6) | 0.9 | (0.5-1.4) |

| Cause of death | SES | Men | | Women | |
|---|---|---|---|---|---|
| | | Mortality rate | 95% UI | Mortality rate | 95% UI |
| | High | 4.3 | (3.3-5.3) | 0.5 | (0.3-0.8) |
| Lip, oral cavity, and pharynx cancer | Low | 19.9 | (16.3-23.6) | 2.4 | (1.7-3.1) |
| | Middle | 18.2 | (14.5-22.2) | 1.9 | (1.3-2.6) |
| | High | 11.0 | (9.1-12.9) | 1.4 | (0.9-1.9) |
| Larynx cancer | Low | 2.2 | (1.7-2.7) | 0.1 | (0.1-0.1) |
| | Middle | 2.0 | (1.5-2.6) | 0.1 | (0.0-0.1) |
| | High | 1.2 | (0.9-1.5) | 0.1 | (0.0-0.1) |
| Esophagus cancer | Low | 19.4 | (11.5-27.5) | 2.7 | (1.5-4.7) |
| | Middle | 17.7 | (10.4-25.4) | 2.1 | (1.2-3.9) |
| | High | 10.8 | (6.6-15.5) | 1.7 | (1.0-2.8) |

SES socioeconomic status, UI uncertainty interval, n.a. not applicable

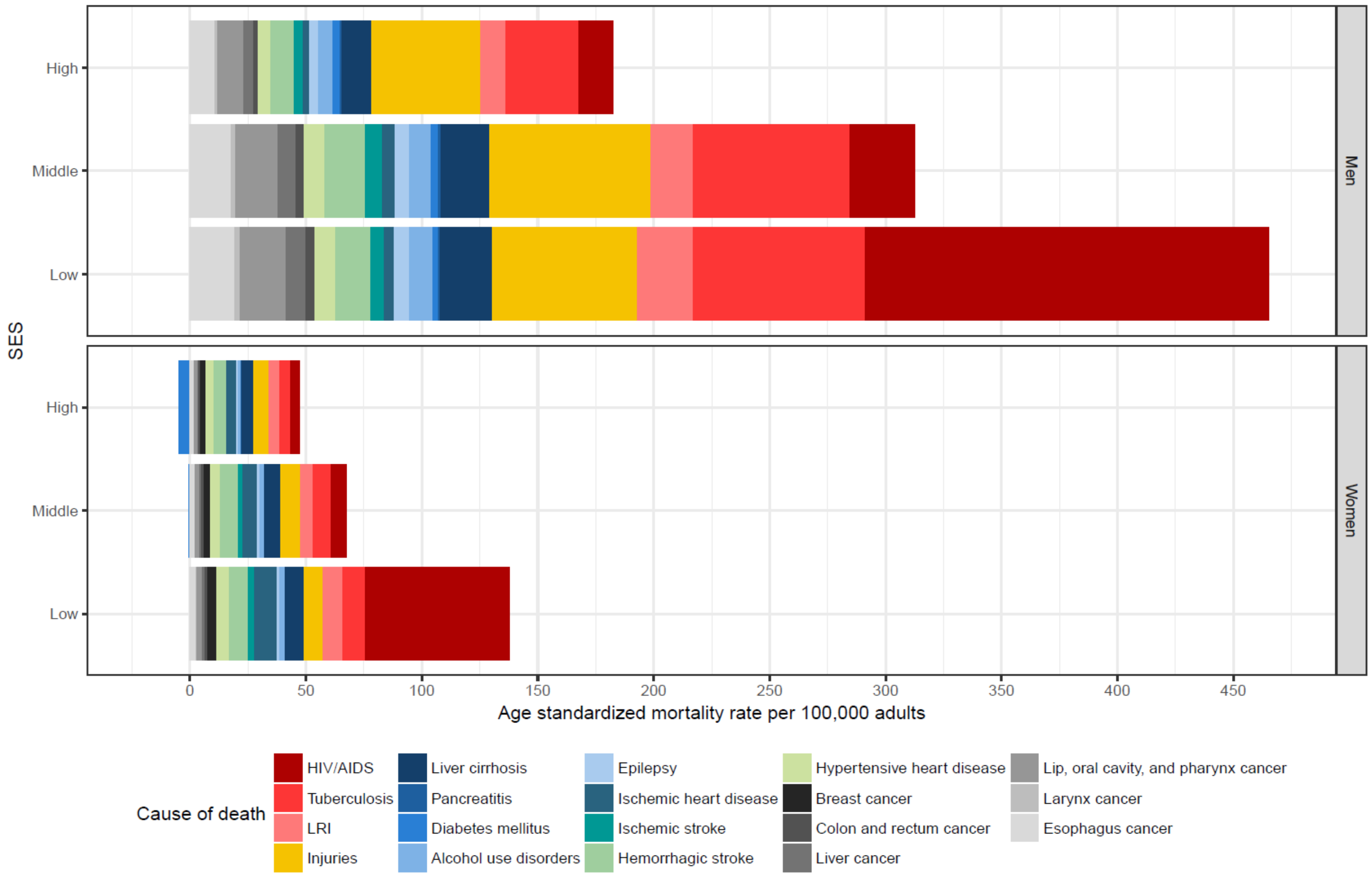


**Figure S2** Sensitivity analysis: Age-standardized mortality rates per 100,000 attributable to alcohol consumption by socioeconomic status (SES) and cause of death among men and women in South Africa, 2015

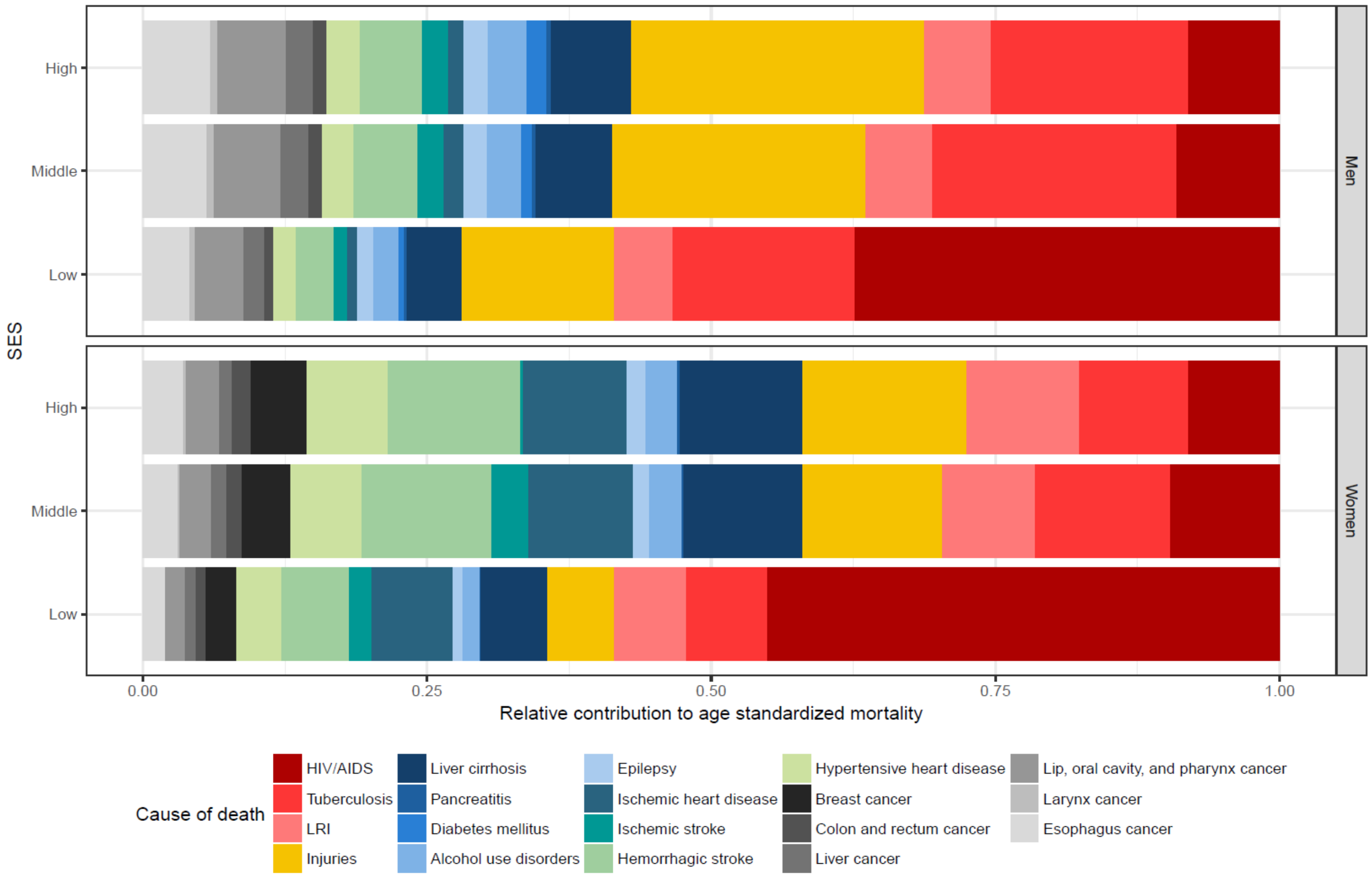


**Figure S3** Sensitivity analysis: Relative contribution of causes of death to the overall age standardized mortality rates by SES for men and women in South Africa, 2015

## References

1. Josse R, Husson F. missMDA: A Package for Handling Missing Values in Multivariate Data Analysis. Journal of Statistical Software 2016, 70(1):1-31.
